# Supplementary material for: Evaluation of different types of face masks to limit the spread of SARS-CoV-2: a modeling study
Source: Sci Rep. 2022 May 23;12:8630. doi: 10.1038/s41598-022-11934-x (PMC9125347; doi:10.1038/s41598-022-11934-x)
Supplement: Supplementary file 1 — Supplementary Information. [file 41598_2022_11934_MOESM1_ESM.pdf]

Supplementary Material for 'Evaluation of Different Types of Face Mask to Limit the Spread of SARS-CoV-2 – A Modeling Study'

B.M. Gurbaxani, A.N. Hill, P. Paul, P.V. Prasad, R.B. Slayton

March 28, 2022

Transmission of SARS-CoV-2 is modeled by a system of ordinary differential equations (ODE) and compartments corresponding to age, disease status, and mask-wearing status. Compartments comprise susceptible (S), exposed (E), pre-symptomatic (P), asymptomatic and undetected (A_u), asymptomatic and detected (A_d), symptomatic (I), recovered (R), deceased (D). The total population is $N = S + E + P + A_u + A_d + I + R$. Initially, $N = 100,000$. Each of these compartments is further stratified by age (16 age groups from the POLYMOD study) and mask-wearing status (yes/no). Thus, each disease compartment is represented by a 16×2 matrix with entries corresponding to the number of individuals of that particular disease status in age group $i = 1, \ldots, 16$ and with mask status $j = 0$ (no mask), 1 (mask). In matrix form, the ODE system is:

$$
\begin{aligned}
\dot{\boldsymbol{S}} &= -q\left\{\boldsymbol{M}((\boldsymbol{I}+p_{\text{rel inf}}\boldsymbol{A}_d)\cdot[\mathbf{1}_{16}\ r\mathbf{1}_{16}]+p_{\text{rel inf}}(\boldsymbol{P}+\boldsymbol{A}_u))/N\begin{bmatrix}1 & 1-w\\ 1-s & (1-w)(1-s)\end{bmatrix}\right\}\cdot\boldsymbol{S}\\
\dot{\boldsymbol{E}} &= q\left\{\boldsymbol{M}((\boldsymbol{I}+p_{\text{rel inf}}\boldsymbol{A}_d)\cdot[\mathbf{1}_{16}\ r\mathbf{1}_{16}]+p_{\text{rel inf}}(\boldsymbol{P}+\boldsymbol{A}_u))/N\begin{bmatrix}1 & 1-w\\ 1-s & (1-w)(1-s)\end{bmatrix}\right\}\cdot\boldsymbol{S}\\
&\quad -\nu_E\boldsymbol{E}\\
\dot{\boldsymbol{P}} &= \nu_E\boldsymbol{E}-\nu_P\boldsymbol{P}\\
\dot{\boldsymbol{A}}_d &= p_{\text{asym}}\,p_{\text{detect}}\,\nu_P\left(\boldsymbol{P}+(\boldsymbol{p}_{\text{inf mask use}}\cdot\boldsymbol{P})\begin{bmatrix}-1 & 1\\ 0 & 0\end{bmatrix}\right)-\nu_A\boldsymbol{A}_d\\
\dot{\boldsymbol{A}}_u &= p_{\text{asym}}\,(1-p_{\text{detect}})\,\nu_P\boldsymbol{P}-\nu_A\boldsymbol{A}_u\\
\dot{\boldsymbol{I}} &= (1-p_{\text{asym}})\,\nu_P\left(\boldsymbol{P}+(p_{\text{know}}\boldsymbol{p}_{\text{inf mask use}}\cdot\boldsymbol{P})\begin{bmatrix}-1 & 1\\ 0 & 0\end{bmatrix}\right)-\nu_I\boldsymbol{I}\\
\dot{\boldsymbol{R}} &= \nu_A(\boldsymbol{A}_d+\boldsymbol{A}_u)+(1-\boldsymbol{p}_{\text{death}})\,\nu_I\boldsymbol{I}
\end{aligned}
$$

Dot superscript denotes derivative with respect to time; central dot $\cdot$ indicates pointwise multiplication of matrices of the same dimension, or of the columns of a matrix by a vector of same dimension.

Compartment durations are specified by a rate ν_J, where J is the compartment. Average duration in a compartment is $1/\nu_J$. These rates model the durations of days exposed (2 days), pre-symptomatic (4 days), asymptomatic (9 days), and symptomatic (9 days). Relative infectiousness of pre-symptomatic and asymptomatic persons compared to symptomatic persons is $p_{\text{rel inf}} = 0.75$. Detection probability of an asymptomatic case is $p_{\text{detect}} = 0.05$ and the proportion of asymptomatic infections is $p_{\text{asym}} = 0.30$. The risk of death for symptomatic persons was inferred from age-specific infection fatality ratios (IFR) via the equation CFR = IFR/$(1-p_{\text{asym}})$, where CFR denotes the case fatality ratio. IFRs are 0.00003 for ages 0-19 years, 0.0002 ages 20-49, 0.005 ages 50-69, 0.054 ages 70 and older. These parameters are based on the September 2020 estimates included in the CDC Pandemic Planning Scenario #5. We further assume that 18.3% of symptomatic individuals know that that they are sick with SARS-CoV-2 and we denote

this fraction by p_{know}. These people put on a mask at higher rates than those who are unaware they are infected.

POLYMOD daily contact rates were obtained from the study by Prem *et al.* [2] The raw matrix $\boldsymbol{C}$ of contact rates was adjusted in the usual fashion to maintain balance (numbers of contacts of age group i with age group j same as that of j with i) by symmetrizing $\boldsymbol{DC}$, where $\boldsymbol{D}$ is the diagonal matrix of age-group population sizes N_i. Accordingly, matrix $\boldsymbol{C}$ is transformed to $\boldsymbol{C}'$ by

$$C'_{ij} = \tfrac{1}{2}(C_{ij} + C_{ji}N_j/N_i)$$

where N_i is the population size of age-group i. Age-distribution of the population was based on American Community Survey (ACS) estimates for the U.S.-population [3]. Matrix $\boldsymbol{C}'$ is, in turn, transformed to give the symmetric matrix $\boldsymbol{M}$ in the ODEs by

$$M_{ij} = \tfrac{N}{2}(C_{ij}/N_j + C_{ji}/N_i) = C'_{ij}/(N_j/N)$$

In this formulation, a typical term in the force of infection (FoI) arising from an age-stratified infectious compartment $J = P, A_d, A_u, I$ of a given mask status is

$$(\boldsymbol{MJ})_i/N = \sum_{k=1}^{m} M_{ik}J_k/N = \sum_{k=1}^{m} C'_{ik}J_k/N_k$$

where $m = 16$ is the number of POLYMOD age groups corresponding to 5-year age bands ranging from younger than 5 years old to 70-74 years old with the oldest age group comprising persons 75 years and older. The FoI is further stratified in the ODEs above depending on mask status. This is implemented by the 2×2 matrix appearing in the differential equations for $\boldsymbol{S}$ and $\boldsymbol{E}$. This matrix governs reductions in the FoI according to source containment efficacy (sce, or s for brevity) and wearer protection efficiency (wpe or w) conferred by the mask type. These combine in four ways depending on the mask status of the infector and infectee (e.g., $1 - \text{wpe}$ and $1 - \text{sce}$ multiply together in the case of transmission by mask wearers to mask wearers). We further assume that symptomatic and detected, asymptomatic people who wear a mask have a lower, daily rate of contact. As contact rates in compartmental models apply to susceptible, not infectious, individuals, we model this as a reduction in infectiousness by a proportion r for both symptomatic and detected, asymptomatic mask-wearers. Specifically, if subscript i indexes age group and superscripts 'none' and 'mask' denote no mask and mask wearing, respectively, expanding the matrix formulation of the ODEs for susceptible individuals gives

$$\begin{aligned}
\dot{S}_i^{\text{none}} &= -q\sum_{k=1}^{m} M_{ik}(K_k^{\text{none}} + (1-\text{sce})K_k^{\text{mask}})/N \times S_i^{\text{none}} \\
&= -q\sum_{k=1}^{m} C'_{ik}(K_k^{\text{none}} + (1-\text{sce})K_k^{\text{mask}})/N_k \times S_i^{\text{none}} \\
\dot{S}_i^{\text{mask}} &= -q(1-\text{wpe})\sum_{k=1}^{m} M_{ik}(K_k^{\text{none}} + (1-\text{sce})K_k^{\text{mask}})/N \times S_i^{\text{mask}} \\
&= -q(1-\text{wpe})\sum_{k=1}^{m} C'_{ik}(K_k^{\text{none}} + (1-\text{sce})K_k^{\text{mask}})/N_k \times S_i^{\text{mask}}
\end{aligned}$$

where

$$\begin{aligned}
\boldsymbol{K}^{\text{none}} &= \boldsymbol{I}^{\text{none}} + p_{\text{rel inf}}(\boldsymbol{P}^{\text{none}} + \boldsymbol{A}_d^{\text{none}} + \boldsymbol{A}_u^{\text{none}}) \\
\boldsymbol{K}^{\text{mask}} &= r\boldsymbol{I}^{\text{mask}} + p_{\text{rel inf}}(\boldsymbol{P}^{\text{mask}} + r\boldsymbol{A}_d^{\text{mask}} + \boldsymbol{A}_u^{\text{mask}}).
\end{aligned}$$

The age-specific vector $\boldsymbol{p}_{\text{inf mask use}}$ (in the set of 7 ODEs specified at the beginning of this supplement) specifies the proportion of detected asymptomatic people who don a mask on learning they are infectious. The corresponding proportion for symptomatic individuals is $p_{\text{know}} \times \boldsymbol{p}_{\text{inf mask use}}$. The 2×2 matrix appearing in the differential equations for $\boldsymbol{A}_d$ and $\boldsymbol{I}$ governs the adoption of masks by asymptomatic individuals when they are detected (the first column corresponds to no mask, the second to mask wearing). In simulations, we assumed that individuals aged 65 years and older adopted masks at one proportion and younger than 65 years at another, lower, proportion (but this can be changed by the user). Expanding the matrix formulation of the ODEs for detected, asymptomatic individuals gives

$$\begin{aligned}
\dot{A}_{d,i}^{\text{none}} &= p_{\text{asym}}\, p_{\text{detect}}\, \nu_P (1 - p_{\text{inf mask use},i}) P_i^{\text{none}} - \nu_A\, A_{d,i}^{\text{none}} \\
\dot{A}_{d,i}^{\text{mask}} &= p_{\text{asym}}\, p_{\text{detect}}\, \nu_P (P_i^{\text{mask}} + p_{\text{inf mask use},i} P_i^{\text{none}}) - \nu_A\, A_{d,i}^{\text{mask}}
\end{aligned}$$

It is assumed that a proportion of the general population (susceptible individuals) wear a mask at the outset and keep it on at all times (or at least when mixing in the population). This proportion can vary by age. We assume that in the general population, 80% of those aged 65 and older, and 60% of the rest, wear a mask (and keep it on indefinitely).

To seed the epidemic, we arbitrarily assumed that there were 10 detected, asymptomatic non-masked individuals in each age group at the outset. Time units were expressed in days. The model was run for 6 months (183 days) with a timestep of 0.25 days using a Runge-Kutta solver in R v.4.0.4 [4] using the package 'deSolve' [5] v.1.29.

The FoI was calibrated to yield a basic reproduction number $R_0 = 2.5$ for the sub-model without mask usage. This yielded the parameter $q = 0.01429$ which represents the probability of a symptomatic infectious person infecting a susceptible person upon contact between them. The reproduction number was calculated as the dominant eigenvalue of the next-generation matrix (NGM) using the method of van den Driessche and Watmough [6]. Computation was facilitated by the R package 'blockmatrix' [7] v.1.0 owing to the sparseness of the matrices involved. Details of this calculation are described further below.

Calculation of R_0

Following [6], we construct matrices F, describing rates at which infectious individuals produce new infections, and V, consisting of all other rates, whose inverse describes average durations in compartments. The ith row and jth column of these matrices is the partial derivative of the right-hand side of the differential equation for compartment i with respect to compartment j, evaluated at the disease free equilibrium (DFE). Only the 5 infected compartment types are considered, namely, E, P, A_d, A_u, I, enumerated by age group and mask status. The basic reproduction number R_0 is given by the dominant eigenvalue of the NGM FV^{-1}.

Matrices F and V are of dimension 160×160 (2 mask statuses $\times$ 16 age groups $\times$ 5 relevant compartment types). However, as new infections only arise from the E compartments, via the

previously described FoI, matrix F is sparse. So too is V as a lot of its sub-blocks are zero or diagonal matrices. Hence, we can construct these matrices in block form. We use the Kronecker product of matrices which we denote by $\otimes$.

The DFE depends on the initial age-specific proportions, $\boldsymbol{p}_{\text{susc mask use}}$, of the general susceptible population wearing a mask. Multiplying the ACS age group proportions pointwise by $\boldsymbol{p}_{\text{susc mask use}}$ and $1 - \boldsymbol{p}_{\text{susc mask use}}$ gives the age-specific proportions of the population with and without masks, respectively. These are multiplied by the hypothetical total population size $N =$ 100,000 to obtain numbers in each stratum.

Vectorizing the 16×2 matrices in the ODEs by stacking columns into a single 32×1 column vector, we have the following constituent matrices for calculating R_0:

$$
\begin{aligned}
F_1 &= \underbrace{\begin{bmatrix}1\\0\\0\\0\\0\end{bmatrix}}_{5\times1} \otimes \left\{ \left(\left(\left\{ \underbrace{\begin{bmatrix}1\\1-w\end{bmatrix}}_{2\times1} \otimes \underbrace{[0\ p_{\text{ri}}\ p_{\text{ri}}\ p_{\text{ri}}\ 1]}_{1\times5} \otimes \underbrace{[1\ 1-s]}_{1\times2} \cdot \underbrace{G}_{2\times10} \right\} \otimes \underbrace{(M \cdot \boldsymbol{p}_{\text{ACS}})}_{16\times16} \right) \cdot \underbrace{\begin{bmatrix}\mathbf{1}_{16} - \boldsymbol{p}_{\text{smu}}\\ \boldsymbol{p}_{\text{smu}}\end{bmatrix}}_{32\times1} \right\} \\
G &= \begin{bmatrix}\mathbf{1}_5^\top & r & \mathbf{1}_3^\top & r\\ \mathbf{1}_5^\top & r & \mathbf{1}_3^\top & r\end{bmatrix}
\end{aligned}
$$

For brevity, p_{ri} denotes $p_{\text{rel inf}}$ above, $\boldsymbol{p}_{\text{ACS}}$ is the ACS population age distribution, $\boldsymbol{p}_{\text{smu}}$ denotes $\boldsymbol{p}_{\text{susc mask use}}$ above, w denotes wpe, s denotes sce, $\mathbf{1}_n$ is the $n \times 1$ vector of 1's, and $\cdot$ represents pointwise multiplication by column. Matrix M is the POLYMOD-derived contact matrix described earlier. It follows from the definition of Kronecker product that F_1 is 160×160. The order of entries in row or column vectors of length 5 corresponds to the compartment types E, P, A_d, A_u, I. Thus, the 5×1 vector on the left represents new infections only arising from compartments of type E (component 1) and not from P, A_d, A_u, I (components 2 to 5) and its occurrence renders F_1 sparse. The 1×5 row vector has components 2-4 as $p_{\text{rel inf}}$, indicating the relative infectiousness of pre-symptomatic and detected and undetected asymptomatic individuals (P, A_d, A_u) compared to symptomatic individuals I (component 5). Row and column vectors of length 2 correspond to mask efficacies. The vector $\boldsymbol{p}_{\text{ACS}}$ represents the population age-distribution and the 32×1 vector on the right denotes the age-specific general population mask wearing proportions.

The matrix F is given by $F = qF_1$, where q is the calibration parameter representing the probability of a symptomatic infectious person infecting a susceptible person upon contact between them.

Matrix V may also be expressed in block form as

$$
V = \begin{bmatrix}
V_{EE} & 0 & 0 & 0 & 0\\
V_{PE} & V_{PP} & 0 & 0 & 0\\
0 & V_{A_dP} & V_{A_dA_d} & 0 & 0\\
0 & V_{A_uP} & 0 & V_{A_uA_u} & 0\\
0 & V_{IP} & 0 & 0 & V_{II}
\end{bmatrix}
$$

where each block is a 32×32 matrix. Theory guarantees V is invertible. As with F, the order of the component types in block rows and columns here is E, P, A_d, A_u, I. The nonzero blocks are

as follows, with parameter notation as given earlier and I_n denoting the $n \times n$ identity matrix:

$$\begin{aligned}
V_{EE} &= \nu_E \, I_{32} \\
V_{PE} &= -\nu_E \, I_{32} \\
V_{PP} &= \nu_P \, I_{32} \\
V_{A_dP} &= -\nu_P \, p_{\text{detect}} \, p_{\text{asym}} B_A \\
\text{where } B_A &= \begin{bmatrix} \text{diag}(1 - \boldsymbol{p}_{\text{inf mask use}}) & 0 \\ \text{diag}(\boldsymbol{p}_{\text{inf mask use}}) & I_{16} \end{bmatrix} \text{ (16} \times \text{16 blocks)} \\
V_{A_dA_d} &= \nu_A \, I_{32} \\
V_{A_uP} &= -\nu_P \, (1 - p_{\text{detect}}) p_{\text{asym}} I_{32} \\
V_{A_uA_u} &= \nu_A \, I_{32} \\
V_{IP} &= -\nu_P \, (1 - p_{\text{asym}}) B_I \\
\text{where } B_I &= \begin{bmatrix} \text{diag}(1 - p_{\text{know}} \boldsymbol{p}_{\text{inf mask use}}) & 0 \\ p_{\text{know}} \text{diag}(\boldsymbol{p}_{\text{inf mask use}}) & I_{16} \end{bmatrix} \text{ (16} \times \text{16 blocks)} \\
V_{II} &= \nu_I \, I_{32}
\end{aligned}$$

Blocks V_{XX} on the leading diagonal correspond to outflows from compartment type X. Off-diagonal blocks V_{XY} correspond to inflows from compartment type Y to compartment type X. The combination of vector $\mathbf{v}$ and matrix B corresponds to adoption of mask (change of mask status) upon asymptomatic detection/symptomatic awareness, according to the age-specific proportion ($\boldsymbol{p}_{\text{inf mask use}}$) who do so. Denoting the dominant eigenvalue of a matrix by ρ, we have

$$R_0 = \rho(FV^{-1}) = q \times \rho(F_1 V^{-1}).$$

Setting baseline $R_0 = 2.5$ (no mask use), we calibrate $q = 2.5 \div \rho(F_1 V^{-1})$. Calibrating to other values of R_0 proceeds similarly.

Computation of symptomatic and asymptomatic detection rates
The percentage of symptomatic and asymptomatic SARS-CoV-2 cases that are detected are not established numbers, but they are suspected to be low given the general detection rate of 16.1% [8, 9]. Both can be estimated with a simple Bayesian calculation, however, given the general detection rate (P(case) in the equation below), the asymptomatic rate of infections of 0.3 [10], and the probability that detected cases are and remain asymptomatic (0.2) or symptomatic (0.8) [11]. For example, the probability that a person becomes a detected case (e.g., through contact tracing efforts) given the person is asymptomatic is given as

$$P(\text{case} \mid \text{asymptomatic}) = P(\text{asymptomatic} \mid \text{case}) \times \frac{P(\text{case})}{P(\text{asymptomatic})}$$

The simple calculation yields a 10.7% detection rate for asymptomatic individuals, and a 18.3% detection rate for those with symptoms. The 18.3% figure appears to be in line with epidemiological estimates as well [12].

References

[1] Worby C.J., Chang H.H. (2020) Face mask use in the general population and optimal resource allocation during the COVID-19 pandemic. Nat Commun 11, 4049. https://doi.org/10.1038/s41467-020-17922-x

[2] Prem K., Cook A.R., Jit M. (2017) Projecting social contact matrices in 152 countries using contact surveys and demographic data. PLoS Comput Biol 13(9): e1005697. https://doi.org/10.1371/journal.pcbi.1005697

[3] U.S. Census Bureau, American Community Survey, TṠ0101, Editor. 2019, U.S. Census Bureau: data.census.gov.

[4] R Core Team (2021). R: A language and environment for statistical computing. R Foundation for Statistical Computing, Vienna, Austria. URL https://www.R-project.org/

[5] Karline Soetaert, Thomas Petzoldt, R. Woodrow Setzer (2010). Solving Differential Equations in R: Package deSolve. Journal of Statistical Software, 33(9), 1-25. URL http://www.jstatsoft.org/v33/i09/ DOI 10.18637/jss.v033.i09

[6] van den Driessche P., Watmough J. Reproduction numbers and sub-threshold endemic equilibria for compartmental models of disease transmission. Math Biosci. 2002 Nov-Dec;180:29-48. doi: 10.1016/s0025-5564(02)00108-6. https://doi.org/10.1016/S0025-5564(02)00108-6 PMID: 12387915.

[7] Emanuele Cordano (2014). blockmatrix: blockmatrix: Tools to solve algebraic systems with partitioned matrices. R package version 1.0. https://CRAN.R-project.org/package=blockmatrix

[8] Alroy KA, Crossa A, Dominianni C, Sell J, Bartley K, Sanderson M, Fernandez S, Levanon Seligson A, Lim S, Wang SM, Dumas SE, Perlman SE, Konty K, Olson DR, Gould LH, Greene SK. Population-Based Estimates of Coronavirus Disease 2019 (COVID-19)-like Illness, COVID-19 Illness, and Rates of Case Ascertainment, Hospitalizations, and Deaths—Noninstitutionalized New York City Residents, March-April 2020. Clin Infect Dis. 2021 Nov 2;73(9):1707-1710. https://doi.org/10.1093/cid/ciab038 PMID: 33458740; PMCID: PMC7929112.

[9] Phipps S.J., Grafton R.Q., Kompas T. Robust estimates of the true (population) infection rate for COVID-19: a backcasting approach. R Soc Open Sci. 2020 Nov 18;7(11):200909. https://doi.org/10.1098/rsos.200909 PMID: 33391791; PMCID: PMC7735365.

[10] Centers for Disease Control and Prevention. COVID-19 Pandemic Planning Scenarios. 2020 September 10, 2020; Available from: https://www.cdc.gov/coronavirus/2019-ncov/hcp/planning-scenarios.html

[11] Buitrago-Garcia D, Egli-Gany D, Counotte MJ, Hossmann S, Imeri H, Ipekci AM, Salanti G, Low N. Occurrence and transmission potential of asymptomatic and presymptomatic SARS-CoV-2 infections: A living systematic review and meta-analysis. PLoS Med. 2020 Sep 22;17(9):e1003346. https://doi.org/10.1371/journal.pmed.1003346 PMID: 32960881; PMCID: PMC7508369.

[12] Smith LE, Potts HWW, Amlôt R, Fear NT, Michie S, Rubin GJ. Adherence to the test, trace, and isolate system in the UK: results from 37 nationally representative surveys. BMJ. 2021 Mar 31;372:n608. https://doi.org/10.1136/bmj.n608 PMID: 33789843; PMCID: PMC8010268.
